# Reproductive function in thyroid cancer survivors: a gender-based perspective on outcomes

**DOI:** 10.1530/EO-26-0047

**Published:** 2026-10-15

**Authors:** Rossella Mazzilli, Francesco Felicetti, Virginia Zamponi, Alice Nervo, Elisa Vaccaro, Giuseppe Defeudis, Emanuela Arvat, Antongiulio Faggiano

**Affiliations:** ^1^ Endocrinology Unit, Department of Clinical and Molecular Medicine, Sapienza University of Rome, Rome, Italy; ^2^ Division of Oncological Endocrinology, Città della Salute e della Scienza Hospital, Turin, Italy; ^3^ Department of Medical Sciences, University of Turin, Torino, Italy; ^4^ Department of Theoretical and Applied Sciences, eCampus University, Novedrate CO, Italy

**Keywords:** thyroid cancer survivors, differentiated thyroid cancer, fertility, hypogonadism, radioiodine therapy, TSH suppression, ovarian reserve, spermatogenesis

## Abstract

**Background:**

Thyroid cancer is one of the most common endocrine malignancies, with an excellent long-term survival rate. As survivorship increases, attention has shifted towards chronic endocrine sequelae, including disturbances of gonadal function and fertility. Thyroid cancer therapies, including thyroidectomy, long-term thyroid-stimulating hormone (TSH) suppressive therapy, radioiodine (RAI), and targeted systemic treatments, may interfere with reproductive health through complex endocrine and gonadal mechanisms.

**Objective:**

This narrative review aims to examine the pathophysiological pathways linking thyroid cancer and its treatments to reproductive health in both men and women.

**Results:**

Thyroidectomy and chronic TSH suppression may alter sex steroid bioavailability and gonadotrophin regulation through changes in thyroid hormone signalling and sex hormone-binding globulin synthesis. RAI therapy exerts dose-dependent gonadotoxic effects, particularly affecting ovarian reserve and spermatogenesis, often in a transient and reversible manner, but potentially accelerating reproductive ageing and damage. Targeted therapies may also contribute to reproductive dysfunction through indirect systemic and endocrine mechanisms; however, data on thyroid cancer patients are still limited.

**Conclusion:**

Thyroid cancer-related reproductive dysfunction may arise from the interaction of endocrine- and treatment-related mechanisms. Although often subclinical, these alterations are biologically relevant and may influence long-term fertility potential. An accurate counselling for fertility management in thyroid cancer survivors appears relevant and highlights areas for future research.

## Introduction

Thyroid cancer is one of the most prevalent endocrine malignancies worldwide, with an increasing incidence in the past 4 decades of about 300%, mainly among young and middle-aged adults and a marked female predominance (1). According to data from the National Cancer Institute, 60% of thyroid cancer cases are diagnosed in women younger than 45 years, with a 5-year relative survival rate of 98.4% for the period 2015–2021 (https://gco.iarc.fr/today/en).

As a consequence of favourable survival rates, especially in differentiated thyroid cancer (DTC), ‘long-term survivorship’ has become a crucial issue. In this regard, a person is considered a ‘cancer survivor’ from the time of diagnosis through the balance of life (https://www.cancer.gov/about-cancer/coping/survivorship) (2). This concept underlines the importance of paying attention not only to mortality but also to chronic endocrine complications and quality-of-life outcomes (https://www.cancer.gov/about-cancer/coping/survivorship).

Among survivorship-related complications, reproductive health represents an important domain. It is well known that thyroid hormones play a critical role in the regulation of the hypothalamic–pituitary–gonadal (HPG) axis, influencing gonadotrophin secretion, sex steroid metabolism, and gametogenesis in both sexes (3, 4, 5). In women, thyroid dysfunction has been associated with menstrual irregularities, anovulation, impaired luteal function, and reduced fertility (5). Both hypothyroidism and hyperthyroidism can interfere with gonadotrophin-releasing hormone (GnRH) pulsatility and alter oestrogen metabolism, potentially affecting ovarian reserve and endometrial receptivity (4, 6). In men, thyroid hormone abnormalities are linked to changes in SHBG concentrations, alterations in total and free testosterone levels, and impaired spermatogenesis, with several effects on semen quality, including impaired sperm concentration, motility, and morphology (3). Autoimmune mechanisms may further contribute to reproductive vulnerability. Thyroid autoimmunity has also been associated with adverse reproductive outcomes even in euthyroid individuals, particularly in women (4). Proposed mechanisms include immune-mediated ovarian dysfunction and systemic inflammatory effects, although causal relationships remain incompletely defined (3, 4).

Consequently, thyroid cancer treatment, including thyroidectomy, long-term levothyroxine (LT4) therapy with TSH suppression, and radioiodine (RAI) treatment, influencing thyroid hormone levels and balance, may exert biological effects on reproductive function (1).

Previously, thyroid cancer has been regarded as a malignancy with minimal reproductive impact, particularly when compared with gonadotoxic chemotherapeutic regimens used in other cancers. However, several studies indicate that thyroid cancer therapies may induce transient or persistent alterations in gonadal function, sex hormone profiles, and fertility potential, with effects that are modulated by sex, age at exposure, and cumulative treatment (7, 8).

Moreover, patients of reproductive age who underwent surgery or other treatments for thyroid cancer reported substantial fertility-related concerns, particularly when future childbearing was desired, highlighting the significant impact of reproductive issues in this population (9).

This narrative review aims to critically evaluate the pathophysiological mechanisms linking thyroid cancer and its treatments to hypogonadism and fertility impairment in both men and women, while also considering the potential bidirectional relationship between reproductive health and thyroid cancer.

## Methods

A comprehensive narrative review of the literature was conducted using PubMed, including studies published up to January 2026. The search strategy included combinations of the terms ‘thyroid cancer’, ‘radioiodine treatment’, ‘female fertility’, ‘male fertility’, ‘female reproductive health’, ‘male reproductive health’, ‘ovarian reserve’, ‘semen parameters’, ‘hypogonadism’, and ‘fertility preservation’.

Prospective and retrospective studies, systematic reviews, meta-analyses, and relevant international guidelines published in English were considered.

For the purposes of this review, evidence obtained specifically from patients with thyroid cancer or thyroid cancer survivors was considered direct evidence. Evidence derived from other cancer populations, mechanistic studies, or clinical settings not specifically involving thyroid cancer was considered indirect evidence and was interpreted cautiously.

For interpretation purposes, endocrine gonadal function, gonadal reserve or gametogenic parameters, and clinically relevant fertility outcomes were considered distinct endpoints.

## Thyroid cancer: classification and management

Thyroid carcinomas arising from the follicular epithelium are classified according to the fifth edition of the World Health Organization (WHO) Classification of Thyroid Tumours (2022) (10). This framework encompasses a spectrum ranging from benign and borderline neoplasms to differentiated thyroid carcinoma (DTC), high-grade differentiated carcinoma (DHGTC), poorly differentiated thyroid carcinoma (PDTC), and anaplastic thyroid carcinoma (ATC), reflecting distinct biological behaviours and prognostic profiles.

Papillary thyroid carcinoma (PTC) accounts for approximately 85% of all thyroid malignancies and is characterised by a predominant lymphatic pattern of spread and frequent multifocality. Although most PTCs exhibit indolent behaviour, specific aggressive variants – such as tall cell, columnar cell, and hobnail – are associated with higher recurrence rates and reduced disease-specific survival. The invasive encapsulated follicular variant shares clinicopathological features with follicular thyroid carcinoma (FTC), particularly in terms of angioinvasion and risk of distant metastasis (11).

FTC represents about 10% of thyroid cancers and typically spreads haematogenously; the extent of capsular and vascular invasion remains the key determinant of prognosis and therapeutic decision-making.

Oncocytic carcinoma, defined by a predominance of oncocytic cells and lacking the nuclear features of PTC, is recognised as a distinct entity due to its different molecular profile and clinical behaviour. High-grade thyroid carcinomas – including DHGTC and PDTC – are characterised by increased mitotic activity and tumour necrosis and carry a prognosis intermediate between well-differentiated carcinomas and ATC. ATC, although rare, remains one of the most aggressive human malignancies, with a median overall survival of only a few months (12).

This histological and biological heterogeneity directly influences the extent of surgery, the indication for radioactive iodine therapy, the need for long-term TSH suppression, and the potential use of systemic treatments. Given that most DTCs occur in individuals with long life expectancy, therapeutic decisions must balance oncological efficacy with the minimisation of long-term treatment-related sequelae.

Surgery represents the cornerstone of treatment for DTC when cytology is suspicious for or diagnostic of malignancy. The extent of surgery, including total thyroidectomy versus lobectomy/haemithyroidectomy, depends on tumour size, multifocality, extrathyroidal extension, nodal involvement, and individual risk stratification (11).

Total thyroidectomy is generally indicated for tumours > 4 cm, bilateral disease, clinically significant nodal metastases, gross extrathyroidal extension, distant metastases, a history of cervical irradiation, or familial disease. Conversely, current guidelines increasingly support a more conservative approach, favouring lobectomy for unifocal, intrathyroidal tumours ≤ 2 cm (cT1N0M0). For tumours measuring 2–4 cm (cT2N0M0), either strategy may be appropriate based on individual risk assessment. Central neck dissection may be prophylactic or therapeutic, whereas lateral neck dissection is reserved for documented nodal metastases. Active surveillance can be considered in carefully selected patients with low-risk microcarcinomas (T1aN0M0) (11) (13).

The extent of initial surgery has important implications not only for recurrence risk but also for subsequent therapeutic burden, including the need for radioactive iodine (RAI) and long-term thyroid hormone therapy.

Following total thyroidectomy, levothyroxine replacement is mandatory, whereas it may not be required after lobectomy. The degree of TSH suppression is tailored according to recurrence risk and response to therapy. For low- and intermediate-risk patients without biochemical or structural evidence of disease, routine TSH suppression below the normal range is not recommended; conversely, sustained suppression may be beneficial in high-risk cases (11).

As thyroid hormone therapy is required for life, the long-term systemic effects of sustained TSH suppression, particularly in younger individuals, represent an important consideration in therapeutic planning.

Approximately three months after surgery, recurrence risk is reassessed by integrating histopathological features with postoperative imaging and biochemical markers (thyroglobulin and anti-thyroglobulin antibodies). Patients are stratified into risk categories that guide the indication for RAI, which is strongly recommended for high-risk patients and selectively considered in intermediate-risk cases based on additional prognostic features. Its use aims to ablate residual thyroid tissue, reduce recurrence risk, and treat iodine-avid metastatic disease (11, 14). While RAI significantly improves disease control in selected patients, its administration entails systemic radiation exposure. Therefore, careful selection is essential to maximise oncological benefit while limiting potential long-term adverse effects.

Dynamic risk stratification continues throughout follow-up, allowing reclassification based on response to treatment. It relies on serial measurement of biochemical markers combined with cervical ultrasound. Assessments are typically performed every 6–12 months initially, then spaced according to estimated risk and response to therapy (11, 15).

The management of structural persistence or recurrence requires an individualised approach based on lesion site, growth kinetics, overall tumour burden, and patient comorbidities. Surgery remains the preferred treatment for resectable locoregional or oligometastatic disease (16). Additional RAI may be effective in iodine-avid metastatic disease, particularly in cases of diffuse pulmonary micrometastases.

In patients with radioiodine-refractory (RAI-R) DTC, therapeutic decisions depend on tumour progression rate, symptom burden, and risk of local complications. About 5% of patients with DTC develop distant metastases, and up to 70% of these eventually become RAI-R. Systemic therapy is reserved for progressive, symptomatic, or life-threatening disease (16, 17).

Multikinase inhibitors (MKIs) constitute the standard treatment for advanced progressive RAI-R DTC. In the absence of actionable molecular alterations, lenvatinib is generally preferred as first-line therapy, whereas cabozantinib is approved following progression on prior systemic treatment. Selective second-generation inhibitors targeting specific driver mutations (e.g. RET fusions) have demonstrated high response rates and improved tolerability profiles (11).

However, systemic therapies are predominantly cytostatic and are associated with chronic toxicities that may significantly impact quality of life, being a major limitation and often necessitating dose reductions or discontinuation. As many patients have prolonged survival even in advanced stages, therapeutic decisions increasingly require a careful balance between disease control and the mitigation of long-term treatment-related toxicity.

## Thyroidectomy and chronic TSH suppression

TSH suppression and subsequent increased thyroid hormone levels may influence the HPG axis through multiple mechanisms (4) (Fig. 1). Thyroid hormones modulate hepatic SHBG synthesis, thereby affecting the balance between total and bioavailable sex steroids.

**Figure 1:**
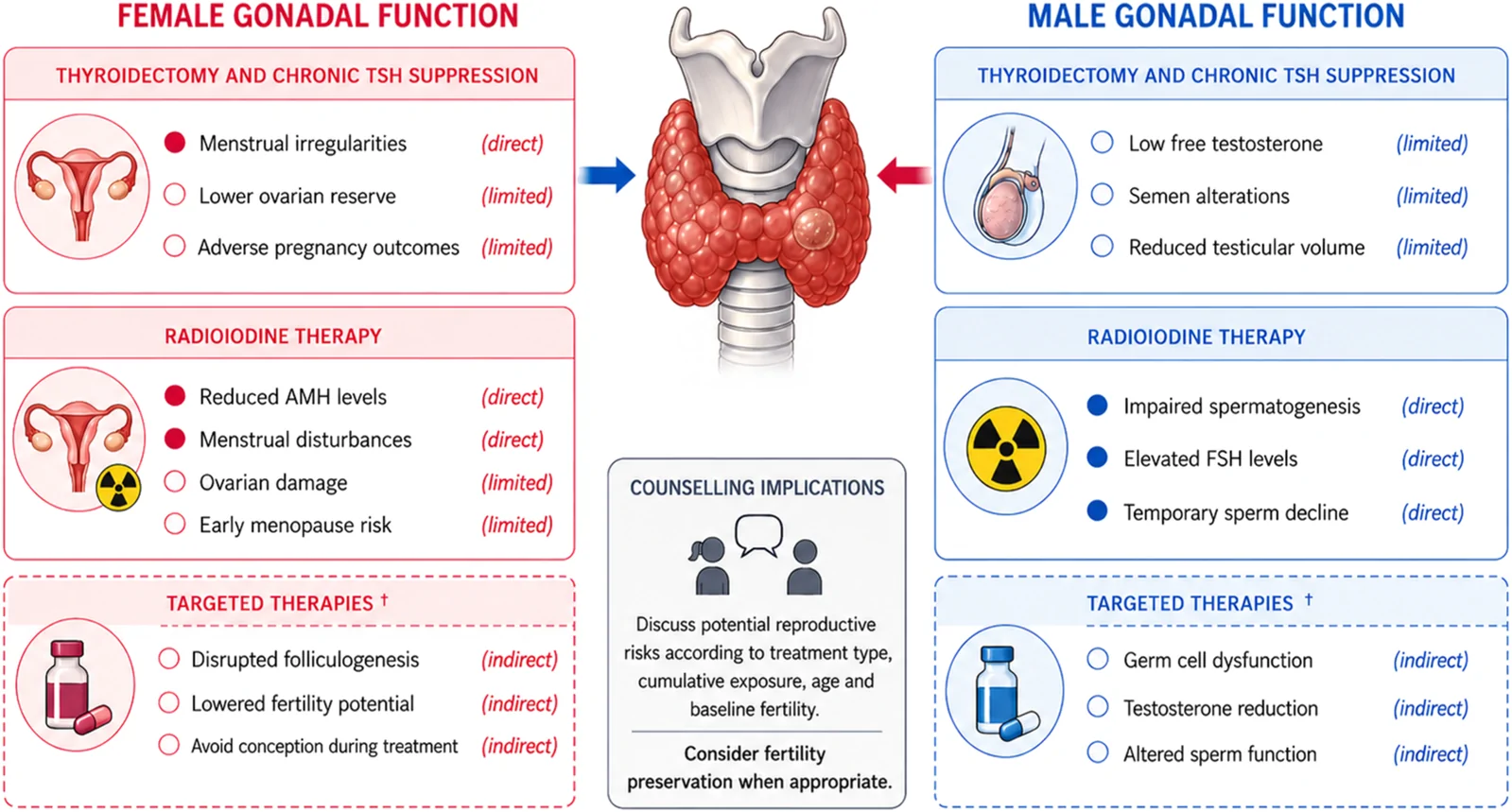
Possible mechanisms underlying the impact of thyroid cancer treatments on gonadal function. Thyroid cancer management, including thyroidectomy with chronic TSH suppression, radioiodine (RAI) therapy, and targeted therapies, may affect the hypothalamic–pituitary–gonadal (HPG) axis in both sexes. In women, these treatments are associated with alterations in ovarian reserve, menstrual function, and reproductive outcomes, mediated by hormonal imbalance, radiation-induced follicular damage, and interference with key signalling pathways regulating folliculogenesis. In men, effects include changes in sex hormone binding, transient or persistent impairment of spermatogenesis, and pathway-specific disruptions induced by targeted therapies. Filled circles indicate findings supported by direct clinical evidence in patients with thyroid cancer or thyroid cancer survivors, whereas open circles indicate findings supported by limited or indirect evidence, including data derived from other oncological populations, mechanistic studies, or clinical settings not specifically involving thyroid cancer.

### Women

In women of reproductive age, long-term TSH suppression has been associated with changes in menstrual cyclicity and subtle alterations in ovarian function, although findings are heterogeneous across studies (18). The interplay between supraphysiological thyroid hormone levels, oestrogen metabolism, and gonadotrophin secretion is complex and may be influenced by age, baseline ovarian reserve, and duration of exposure (18).

In patients with previous DTC, during pregnancy, TSH should be managed at the same target as determined preconceptionally, and TSH levels should be monitored every 4–6 weeks (19).

Furthermore, a study conducted on 212 singleton pregnancies with thyroid cancer under LT4 suppression and 35.641 controls revealed a higher risk of late miscarriage (OR: 7.166; 95% CI: 1.521–33.775; *P* = 0.013), although this association was not statistically significant after adjustment for maternal TPOAb. The study also reported greater gestational weight gain in the first group compared with controls (14 vs 13 kg; *P* < 0.001) (20). Another study exploring obstetric outcomes in 204 women with previous DTC found a higher risk of fetal growth restriction (OR: 5.21; 95% CI: 1.21–22.55) compared with controls. Interestingly, the rates of pregnancy termination and iatrogenic preterm birth decreased over successive 6-year periods (21).

On the contrary, a study conducted on pregnant women, of whom 154 had a history of thyroid cancer under LT4 suppression and 308 were controls, revealed no differences in adverse obstetric outcomes, except for a higher risk of abnormally invasive placentation (22).

On the other hand, thyroid hormones play a critical role in fetal development, and the deficiency during critical periods of brain development can adversely affect obstetric outcomes and may produce irreversible brain damage and neurocognitive delay (23).

### Men

In men, LT4-induced increases in SHBG may lead to elevated total testosterone levels while reducing free testosterone, potentially contributing to symptoms of functional hypogonadism despite apparently normal androgen concentrations (24). Furthermore, hyperthyroidism affects Sertoli cell proliferation, with a reduction of testis volume and negative effects on spermatogenesis (24). Specifically, this condition has been associated with reduced semen volume and low sperm concentration, motility, and impaired sperm morphology (25).

## Radioiodine therapy

Although RAI is selectively taken up by thyroid tissue via the sodium-iodide symporter, gonadal exposure occurs through systemic circulation, resulting in low but measurable radiation doses to the ovaries and testes (4, 7) (Fig. 1).

### Women

Ovarian tissue is particularly sensitive to ionising radiation due to the non-renewable pool of primordial follicles. RAI exposure may induce follicular DNA damage, microvascular injury, and oxidative stress, leading to accelerated follicular atresia, as well as a fetal thyroid disruption if used during pregnancy (4). Anti-Müllerian hormone (AMH), a marker of ovarian reserve, has therefore been widely used to assess subclinical ovarian damage following RAI therapy (26, 27).

A recent systematic review and meta-analysis explored 22 studies on this topic and found menstrual irregularities in the first year after RAI in 12–31% of the patients (27). Specifically, 8–16% of patients reported amenorrhoea. Furthermore, the median age of menopause was lower in DTC patients receiving RAI compared with women who did not receive this treatment (49.5 vs 51 years, respectively, *P* < 0.001). Comparing AMH concentrations before and 1 year after RAI, a significant mean reduction of 1.50 ng/mL in AMH levels was observed. The authors concluded the study with recommendations regarding counselling for patients about the possible effects of 131I and the importance of a multidisciplinary team (27).

Several observational studies have reported a significant decline in ovarian reserve marker levels after RAI administration, particularly in women treated at a younger age or receiving higher cumulative activities (28). While partial recovery of AMH has been observed over time in some cohorts, longitudinal studies suggest that RAI may be associated with a persistent reduction in ovarian reserve markers in a subset of women. However, a decline in AMH should not be interpreted as equivalent to premature ovarian insufficiency, irreversible ovarian failure, or infertility, and the available evidence does not consistently demonstrate an adverse effect of RAI on future fertility or live birth rates (27).

Accordingly, the American Thyroid Association guidelines recommended that women of childbearing age receiving RAI should have a negative screening evaluation for pregnancy prior to RAI administration and avoid pregnancy for 6–12 months after receiving RAI. Furthermore, RAI should not be given during breastfeeding or pumping for at least 3 months (26). However, the European Thyroid Association guidelines recommended that RAI treatment for thyroid cancer should not be delayed for fertility reasons, but women, particularly those older than 35 years, should be informed about the impact of RAI on the live birth rate (29). On the other hand, the latest guidelines for adult patients with DTC state that RAI has not been shown to impact future fertility (Good Practice Statement) (11).

Finally, a study was conducted on 78 pregnancies in 66 women, who previously underwent RAI (3.75–5.5 GBq, mean 4.64 GBq) obtained at least one year after ablative therapy (mean of 30 months), and found that this treatment was not related to a higher risk of malformations (8).

The clinical significance of post-RAI changes in ovarian reserve should, therefore, be interpreted in light of age, baseline reproductive status, cumulative RAI exposure, and reproductive plans.

### Men

A recent systematic review and meta-analysis evaluated the impact of RAI on male reproductive health (7). An elevation in follicle-stimulating hormone (FSH) appears critical after 12 months, mainly influenced by patient age and dose-dependent on I-131 administered. On the contrary, luteinising hormone (LH) was not significantly affected, showing that Leydig cell function, and thus testosterone production, appears to be relatively preserved. The impact of RAI on semen parameters has been less extensively investigated (30, 31)

Specifically, a longitudinal prospective study conducted on 40 young DTC patients evaluated semen parameters before a single 131I ablative treatment and after 3 and 13 months and found a transient but significant reduction of sperm concentration and percentage of normal morphology, which returned to baseline values at 13 months (30). Similarly, another prospective cohort study on a smaller population (18 men with DTC) found a significant reduction of sperm concentration after 3 months, which returned to baseline values after 12 months; sperm motility and morphology were not affected (31).

These findings suggest a predominantly transient impairment of spermatogenic function, with relatively preserved Leydig cell function in most patients.

The American and European Thyroid Association guidelines recommend that men receiving cumulative RAI activities greater than 400 mCi should be counselled on potential risks of infertility and, when multiple doses might be necessary, sperm banking should be offered (11, 29). In addition, men who desire fertility and need to undergo RAI should wait at least 120 days before conception, including through assisted reproductive technology (26, 29).

## Targeted therapies and systemic endocrine interference

Evidence regarding the reproductive effects of TKIs in thyroid cancer survivors is scarce. Most available data derive from other malignancies and should, therefore, be considered indirect evidence when extrapolated to patients with thyroid cancer (Fig. 1).

### Women

Direct clinical evidence on the long-term effects of TKIs on female reproductive function in thyroid cancer patients is currently lacking. Available mechanistic and clinical data from other oncological settings suggest that these agents may interfere with pathways involved in ovarian function (32). In particular, blockade of c-KIT and platelet-derived growth factor receptor (PDGFR)-α disrupts follicular development, reducing proliferation and increasing apoptosis in ovarian cells, ultimately lowering follicle reserve. Src-family kinases also play a role in oocyte maturation. A case report showed that imatinib reaches follicular fluid at concentrations comparable to plasma levels, with no detectable drug or metabolites after clearance (33).

However, planning of pregnancy is suggested to avoid conception during TKI treatment (34).

### Men

In men, the biological rationale is mainly related to the inhibition of key signalling pathways involved in spermatogenesis. In particular, the stem cell factor (SCF)/c-KIT axis plays a crucial role in germ cell survival, differentiation, Leydig cell function, and testosterone synthesis, while downstream pathways such as PI3K are essential for germ cell maturation and Sertoli cell support. In addition, TKIs may interfere with meiotic processes and alter sperm maturation and function by affecting phosphorylation-dependent mechanisms, calcium flux, and acrosome reaction (34, 35).

A recent comparative analysis of the effects of TKIs and ICIs versus conventional chemotherapy on spermatogenesis and testicular endocrine function across heterogeneous malignancies (including thyroid cancer, acute lymphocytic leukaemia, lung, kidney) revealed that TKIs were associated with a modest impact on Leydig cell function and a relatively limited impairment of spermatogenesis, whereas chemotherapy resulted in the most pronounced gonadal toxicity, including a significantly higher incidence of azoospermia (36). Notably, dasatinib and imatinib showed no significant differences from controls in sperm parameters, gonadotrophins, testosterone levels, or testicular volume. In contrast, exposure to gefitinib, sorafenib, or sunitinib was associated with reduced sperm concentration, increased FSH, and decreased testosterone levels, suggesting a differential, drug-specific effect within the TKI class. Pregnancy was achieved in approximately 50% of cases (34).

## Fertility treatment and thyroid cancer

Infertility represents a global health concern involving both male and female populations (37).

Beyond the potential effects of thyroid cancer and its treatments on reproductive function, the relationship between reproductive health and thyroid cancer may also be considered from the opposite perspective, namely whether fertility treatments may be associated with subsequent thyroid cancer risk (38) (Table 1).

**Table 1:** Fertility treatments and risk of thyroid cancer.

Population	Exposure	Main findings	Limits and key considerations
Women	Clomiphene citrate	Increased risk of thyroid cancer (RR up to 3.09 in parous women)	Only cohort studies Possible dose- and parity-dependent effect
	Progesterone	Increased risk (RR: ∼ 10.14)	Only single study Requires confirmation in larger cohorts
	Gonadotrophins/hCG/GnRH	No consistent association with thyroid cancer	Heterogeneous treatment exposure
	Assisted reproductive treatments (overall)	Slight, non-significant increase in thyroid cancer risk	Long-term follow-up studies Potential surveillance bias
	–	Recommendation for patient counselling (ASRM)	Guideline-based Patients should be informed of potential risks
Men	Fertility treatments	No direct evidence available	Major knowledge gap
	Male infertility (baseline condition)	Increased risk of thyroid cancer (HR: 1.52; 95% CI: 1.01–2.30)	Shared pathogenic mechanisms suspected
	Non-obstructive azoospermia	Possible shared molecular pathways with thyroid cancer	

ASRM, American Society for Reproductive Medicine; hCG, human chorionic gonadotrophin; GnRH, gonadotrophin-releasing hormone.

### Women

A study conducted on a cohort of 54,362 women who underwent fertility treatment (including clomiphene, gonadotrophins, hCG, and GnRH) revealed a total of 29 thyroid cancers during follow-up, with an increased risk among patients using clomiphene (RR = 2.28) or progesterone (RR = 10.14). Specifically, the risk was higher in the case of clomiphene (RR = 3.09; 95% CI: 1.21–7.88) in parous women (39).

Subsequently, a study including 285,332 women who underwent fertility treatment, with a median follow-up of 30.0 years, found 91 colorectal cancers, 84 lung cancers, 55 thyroid cancers, and 70 melanomas among study subjects. Clomiphene citrate, used by 38.1% of patients, appears to be related to an increased but non-significant risk of thyroid cancer (40).

In 2024, the American Society for Reproductive Medicine (ASRM) highlighted the importance of counselling, recommending that women should be informed of the higher risk of thyroid cancer associated with clomiphene treatment (41).

### Men

Data on the risk of thyroid cancer in men receiving treatment for fertility are not available. However, subfertile men appear to have a higher risk of thyroid malignancies, in comparison with controls (HR 1.52, 95% CI: 1.01–2.30) (42). On the other hand, men with non-obstructive azoospermia share several pathogenic causes of the development of thyroid cancers, including abnormal glycoprotein hormone alpha subunits and mutations of thyroid receptors (43).

## Thyroid cancer survivors and sexual health

Sexual dysfunction has been reported in cancer survivors of both sexes, including reduced libido, arousal difficulties, and impaired sexual satisfaction (44). These manifestations appear to relate to hormonal imbalance, fatigue, and emotional distress, together with gonadal dysfunction (44).

In men, alterations in free testosterone availability due to increased SHBG under LT4 suppressive therapy may contribute to sexual symptoms despite normal total testosterone levels (3, 45). In women, subtle disturbances in oestrogen–progesterone balance and ovarian reserve may intersect with psychological factors to influence sexual well-being (3).

## Clinical and genetic counselling

Reproductive counselling should be considered in patients of reproductive age with thyroid cancer, particularly when treatment may include RAI or repeated systemic therapies. Before treatment, reproductive plans and potential treatment-related gonadal risks should be discussed, taking into account patient age, baseline reproductive status, and disease severity.

In women, counselling before RAI should include the recommended interval between treatment and conception, as well as the possibility of transient menstrual disturbances and changes in ovarian reserve markers (including AMH levels). Fertility preservation may also be considered according to individual clinical characteristics (11, 26, 27).

In men, the possibility of transient impairment of spermatogenesis should be discussed, particularly when repeated or high cumulative RAI activities are expected. Sperm cryopreservation may be considered before treatment in selected patients, especially when cumulative RAI exposure is likely to be substantial (11, 29).

For thyroid cancer survivors who require ART, management should focus on the appropriate timing of fertility treatment in relation to oncological therapy, optimisation of thyroid function before conception, and individualised assessment of reproductive status (29).

Genetic factors play a central role in specific thyroid cancer subtypes, particularly medullary thyroid carcinoma (MTC), which may occur sporadically or as part of multiple endocrine neoplasia type 2 (MEN2) syndromes caused by germline RET mutations (46). RET mutations do not appear to directly impair gonadal function; however, the endocrine burden associated with prophylactic thyroidectomy, chronic LT4 therapy, and potential multiglandular involvement may indirectly affect reproductive health (46). On the other hand, unlike DTC, where reproductive concerns are primarily related to treatment-induced endocrine alterations, hereditary MTC raises questions regarding the implications of reproduction in mutation carriers, and the chance to perform assisted reproductive technology combined with preimplantation genetic testing (47). In this context, the American Thyroid Association guidelines recommended that patients with MEN 2 be informed about the availability of this test (48).

Overall, counselling should be individualised and preferably involve a multidisciplinary approach including endocrinologists, oncologists, reproductive medicine specialists, and, when appropriate, genetic counselling.

## Conclusions

Thyroid cancer and its treatments may affect reproductive health through distinct effects on gonadal endocrine function, ovarian reserve or spermatogenic function, and clinically relevant fertility outcomes.

Chronic TSH suppression, radioiodine exposure, and systemic targeted therapies converge on the HPG axis through distinct yet interconnected pathways. These effects are modulated by sex, age, baseline endocrine status, and cumulative treatment effect, resulting in heterogeneous reproductive outcomes among survivors. Future research should prioritise longitudinal, sex-specific investigations integrating hormonal profiling, gonadal biomarkers, and reproductive outcomes. Such efforts are essential to refine the biological understanding of thyroid cancer-related reproductive dysfunction and to inform a more nuanced interpretation of fertility outcomes in this growing survivor population.

## Declaration of interest

The authors declare that there is no conflict of interest that could be perceived as prejudicing the impartiality of this work.

## Funding

This work did not receive any specific grant from any funding agency in the public, commercial, or not-for-profit sector.

## Author contribution statement

RM and AF conceived the study. RM, FF, VZ, and EV wrote the paper. AN, GD, EA, and AF revised the manuscript.
